# Actin Depolymerization Factor ADF1 Regulated by MYB30 Plays an Important Role in Plant Thermal Adaptation

**DOI:** 10.3390/ijms24065675

**Published:** 2023-03-16

**Authors:** Lu Wang, Jianing Cheng, Shuangtian Bi, Jinshu Wang, Xin Cheng, Shihang Liu, Yue Gao, Qingkuo Lan, Xiaowei Shi, Yong Wang, Xin Zhao, Xin Qi, Shiyong Xu, Che Wang

**Affiliations:** 1 College of Bioscience and Biotechnology, Shenyang Agricultural University, Shenyang 110866, China; wllhappy1314@163.com (L.W.); cjn960705@stu.syau.edu.cn (J.C.); 2019200041@stu.syau.edu.cn (S.B.); wangjinshu@stu.syau.edu.cn (J.W.); 2020200044@stu.syau.edu.cn (X.C.); 2020220187@stu.syau.edu.cn (S.L.); gyue1217@syau.edu.cn (Y.G.); 2 Institute of Germplasm Resource and Biotechnology, Tianjin Academy of Agricultural Sciences, Tianjin 300384, China; lanqingkuo@163.com (Q.L.); shxw1998@163.com (X.S.); wytaas@126.com (Y.W.); zhaoxin2008999@126.com (X.Z.); 13121253699@163.com (X.Q.); whui175@163.com (S.X.); 3 State Key Laboratory of Vegetable Biobreeding, Tianjin Academy of Agricultural Sciences, Tianjin 300392, China

**Keywords:** ADF1, MYB30, actin filaments, thermal adaptation, *Arabidopsis*, Chinese cabbage

## Abstract

Actin filaments are essential for plant adaptation to high temperatures. However, the molecular mechanisms of actin filaments in plant thermal adaptation remain unclear. Here, we found that the expression of Arabidopsis actin depolymerization factor 1 (*AtADF1*) was repressed by high temperatures. Compared with wild-type seedlings (WT), the mutation of *AtADF1* and the overexpression of *AtADF1* led to promoted and inhibited plant growth under high temperature conditions, respectively. Further, high temperatures induced the stability of actin filaments in plants. Compared with WT, *Atadf1-1* mutant seedlings showed more stability of actin filaments under normal and high temperature conditions, while the *AtADF1* overexpression seedlings showed the opposite results. Additionally, AtMYB30 directly bound to the promoter of *AtADF1* at a known AtMYB30 binding site, AACAAAC, and promoted the transcription of *AtADF1* under high temperature treatments. Genetic analysis further indicated that AtMYB30 regulated *AtADF1* under high temperature treatments. Chinese cabbage ADF1 (BrADF1) was highly homologous with AtADF1. The expression of *BrADF1* was inhibited by high temperatures. *BrADF1* overexpression inhibited plant growth and reduced the percentage of actin cable and the average length of actin filaments in *Arabidopsis*, which were similar to those of *AtADF1* overexpression seedlings. AtADF1 and BrADF1 also affected the expression of some key heat response genes. In conclusion, our results indicate that ADF1 plays an important role in plant thermal adaptation by blocking the high-temperature-induced stability of actin filaments and is directly regulated by MYB30.

## 1. Introduction

High temperature affects the growth and development of plants and results in physiologic changes and intracellular signaling responses in plants [1,2]. In order to survive and grow in high temperature environments, plants develop a series of developmental regulations and mechanisms to enhance their thermal adaptability, including short-term adaptation mechanisms and long-term morphological adaptations [3,4,5,6].

Plants respond to high temperatures by promoting membrane fluidity, thus inducing Ca^2+^ influx and cytoskeletal reorganization [2,7,8]. Actin filament, one of the most important members of the cytoskeleton, plays an important role in cell morphogenesis and signal processes during plant development and high temperature responses [2,4,8,9]. Previous studies have reported that the actin filaments depolymerized at high temperature (41 °C) but were bundled at medium–high temperature (35 °C) in the root epidermal cells of *Arabidopsis* [10]. In addition, the heat-denatured actin filaments are related to heat shock proteins (HSPs) [11,12]. In animal cells, for example, HSP20 regulates actin cytoskeletal dynamics by competing with the actin depolymerizing protein cofilin [11]. Additionally, human HSP27 prevents the aggregation of thermally denatured F-actin by forming soluble complex with denatured actin [12]. However, the molecular mechanisms of actin filaments in plant thermal adaptation are still unknown.

The organization and dynamics of actin filaments are regulated by actin-binding proteins, which usually possess different functions in regulating actin filaments [9,13,14]. Actin depolymerization factor (ADF)/cofilin family proteins regulate microfilament dynamics by promoting microfilament severing and/or depolymerization [13,15], which affects cell morphogenesis, thus contributing to plant growth and development. Recent studies have shown that ADFs play important roles in plant processes such as pollen tube germination and elongation, hypocotyl elongation, stomatal aperture, leaf development, and the abiotic stress response [16,17,18,19,20,21]. In smooth cordgrass, *ADF* is induced to have up-regulated expression in roots but down-regulated expression in leaves under heat stress [22]. The transcription level of *OsADF3* in heat-tolerant rice varieties is higher [23]. As a member of subclass I of the Arabidopsis ADF family, ADF1 is highly expressed in the whole plant [24,25] and participates in the elongation of hypocotyls and the growth of abnormal cotyledons [21]. In addition, *ZmADF1* exhibits high expression under high temperatures [26], suggesting that *ADF1* might be involved in plant growth and development under high temperatures.

MYB transcription factors play an important role in regulating development and responding to abiotic stress [27]. *AtMYB30* belongs to a R2R3-MYB type transcription factor that participates in various stress responses [28]. *AtPIF4*, a target gene of AtMYB30, is a temperature-responsive transcription regulator and plays an important role in the daytime temperature sensing of phyB in *Arabidopsis* [29,30]. Besides, it has been reported that AtMYB30 is partially mediated by AtANN1 and AtANN4 to respond to heat via the calcium signal [31]. These results illustrated that AtMYB30 is involved in responding to heat via several pathways.

Chinese cabbage (*Brassica rapa* L. ssp. *pekinensis*) is one of the main leaf vegetables, widely distributed in China, Korea, Japan, and East Asia [32]. Medium–high temperature is one of the most important factors affecting the growth of plant leaves [33,34]. Many differentially expressed genes have been identified in heat-sensitive and heat-tolerant varieties of non-heading Chinese cabbage [35,36,37,38]. Among them, microRNAs play significant roles in the heat tolerance of flowering Chinese cabbage by mediating the expression of target genes at the post-transcriptional level [33,35]. Additionally, HSPs and HSFs are involved in the heat-shock regulatory network of Chinese cabbage [36]. However, the roles of actin filaments in Chinese cabbage thermal adaptation remain unknown.

In this study, we found that AtADF1 participated in thermal adaptation by affecting the organization of actin filaments. In addition, AtMYB30 directly binds to the promoter of *AtADF1* to promote its expression under high temperatures. The phenotype of *AtMYB30-AtADF1* double-gene genetic materials further indicated that AtADF1 was a downstream member of AtMYB30 in plant thermal adaptation. Furthermore, we confirmed that Chinese cabbage BrADF1, which is highly homologous to AtADF1, possessed a complete F-actin domain, modified actin filaments, and played the same role as AtADF1 in regulating F-actin dynamics and plant thermal adaptation. In conclusion, our results illustrated that ADF1, regulated by MYB30, is an important regulator of actin filaments in the process of plants adapting to high temperatures in *Arabidopsis*, and the same mechanism is also involved in Chinese cabbage thermal adaptation.

## 2. Results

### 2.1. The Expression of AtADF1 Is Down-Regulated under High Temperature

**Figure 1:**
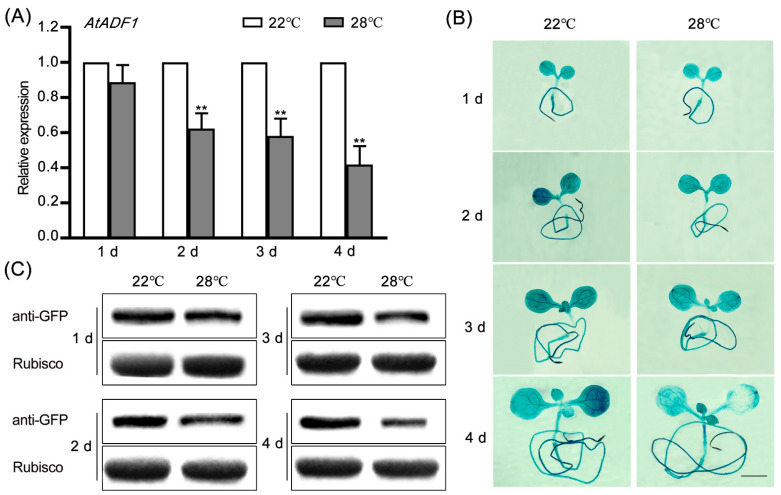
The expression and promoter activity of *AtADF1* are inhibited by high temperatures. (**A**) The relative expression of *AtADF1* in 3-day-old wild type seedlings treated at 28 °C for 1, 2, 3, and 4 d was determined by RT-qPCR. *18S* was used as an internal control. Values are means ± SD from three independent replicate experiments (Student’s *t*-test, ** p < 0.01). (**B**) The expression pattern of *AtADF1* was revealed by GUS staining of *pADF1:GUS* transgenic plants under high temperature. Scale bars = 0.25 cm. (**C**) Western blots using proteins were extracted from *AtADF1*-GFP seedlings incubated at high temperatures. Rubisco was used as a loading control.

To confirm whether AtADF1 responds to high temperature, the expression of AtADF1 was detected at transcription and translation levels. Quantitative real time PCR (RT-qPCR) analysis showed that the expression of *AtADF1* was down-regulated by high temperature treatments (Figure 1A). Further GUS staining analysis verified these results (Figure 1B). In addition, the expression of AtADF1 at the protein level was reduced by high temperature treatments (Figure 1C). These data demonstrated that AtADF1 is down-regulated at transcription and translation levels by high temperature, suggesting that AtADF1 is responsive to high temperature.

### 2.2. AtADF1 Is a Negative Regulator of Plant Growth and Actin Filament Stability under High Temperature

**Figure 2:**
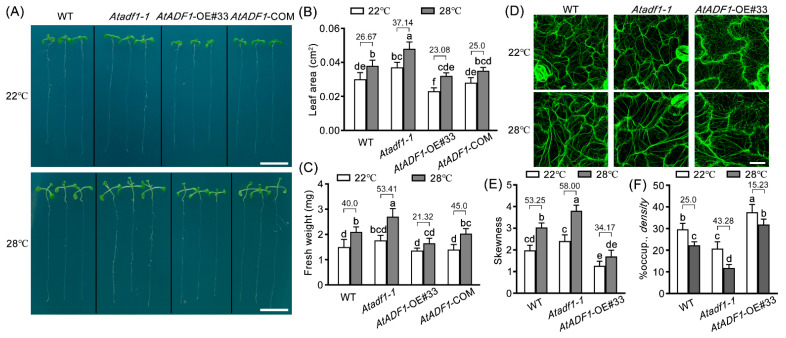
*AtADF1* negatively regulates plant growth and inhibits the stability of actin filaments under high temperature. (**A**) The thermal adaptation of WT, *Atadf1-1*, *AtADF1*-OE#33, and *AtADF1*-COM seedlings was visualized. Three-day-old seedlings of WT, *Atadf1-1*, *AtADF1*-OE#33, and *AtADF1*-COM were transferred into a 28 °C chamber for four days. Scale bar = 1 cm. (**B**,**C**) Leaf area and fresh weight of WT, *Atadf1-1*, *AtADF1*-OE#33, and *AtADF1*-COM seedlings at normal (22 °C) and high temperature (28 °C). At least 60 leaves from 30 seedlings were examined in (**B**) and at least 300 seedlings were examined in (**C**) for per genotype and treatment. Values are mean ± SD from three independent replicate experiments. All of statistical analysis use one-way ANOVA followed by a Tukey’s post-hoc test. Significant differences were indicated by different lowercase letters. (**D**) Representative images of the actin filaments in leaf pavement cells of WT, *Atadf1-1,* and *AtADF1*-OE#33 seedlings grown at 22 °C and 28 °C. Scale bar = 25 μm. (**E**,**F**) The skewness and average filament percentage of occupancy, or density of actin filaments were measured on images shown in (**D**). Values are means ± SD (n > 300 images from at least 30 seedlings for per genotype and treatment). All of statistical analysis use one-way ANOVA followed by a Tukey’s post-hoc test. Significant differences were indicated by different lowercase letters. The number on the column in (**B**,**C**,**E**,**F**) is the percentage of increase or decrease between normal and high temperature conditions.

The 3-d-old WT, *Atadf1-1*, *AtADF1*-OE#33, and *AtADF1*-COM seedlings grown at 22 °C were transferred to 22 °C (control treatment, normal condition) or 28 °C (high temperature treatment) for 4 days, and then the phenotypes were observed (Figure 2A). Under the same condition, *Atadf1-1* mutant seedlings had a larger leaf area and a heavier fresh weight than WT, but *AtADF1*-OE#33 seedlings had the opposite phenotype (Figure 2A–C). Under the high temperature condition, the leaf area and fresh weight of all genotype seedlings were significantly increased, but compared with WT, *Atadf1-1* had a higher increase rate of leaf area and fresh weight, and *AtADF1*-OE#33 exhibited the opposite results (Figure 2B,C). These results indicated that high temperatures promote the growth of seedlings for a period of time and that *AtADF1* plays an important role in regulating plant growth under high temperatures.

The actin filament organization of WT, *Atadf1-1,* and *AtADF1*-OE#33 seedlings fused with *f*ABD2-GFP (a green fluorescent protein (GFP)-based marker used to reveal actin filaments) were observed to explore the function of AtADF1 on actin filaments under high temperature conditions. The skewness (the extent of actin filament bundling) and density (the occupancy ratio of actin filaments) were used to quantify the actin filaments. High temperatures induced the stabilization of actin filaments. Compared with WT, the actin filaments of *Atadf1-1* showed more obvious stability with the increased skewness and reduced density under high temperature conditions, while *AtADF1*-OE#33 showed the opposite results (Figure 2D–F). These results demonstrated that AtADF1 inhibited high-temperature-induced polymerization of actin filaments.

### 2.3. The Expression of AtADF1 Is Positively Regulated by AtMYB30 under High Temperature and AtADF1 Is a Direct Target Gene of AtMYB30

**Figure 3:**
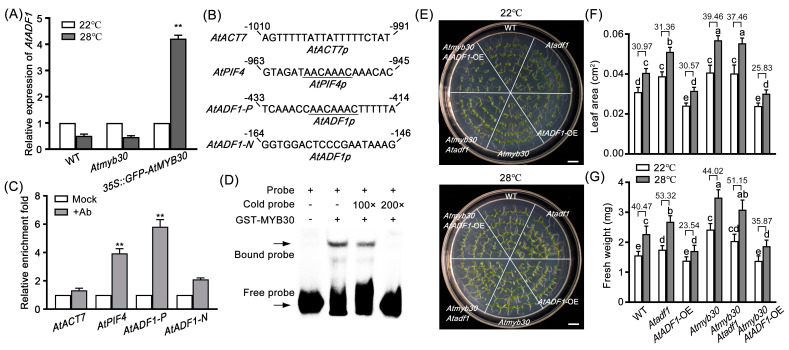
AtMYB30 directly binds to the *AtADF1* promoter and positively regulates the expression of *AtADF1* under high temperature. (**A**) The relative expression of *AtADF1* in 3−day−old WT, *Atmyb30,* and *AtMYB30* OE seedlings treated with 28 °C for 4 d was determined by RT-qPCR. Data show mean values ± SD from three independent replicates (Student’s *t*-test, ** *p* < 0.05). (**B**) MYB−binding site AACAAAC in the *AtADF1* promoter. (**C**) ChIP−qPCR analysis indicates that AtMYB30 is associated with the *AtADF1* promoter in vivo. *AtADF1*−*N* and *AtACT7* are as negative controls, and *AtPIF4* is as a positive control. Data show mean values ± SD from three independent replicates. (Student’s *t*-test, ** p < 0.01). (**D**) EMSA assay of the interaction between AtMYB30 and *AtADF1* promoter. The arrow indicates the bands resulting from GST−MYB30 binding to *AtADF1* promoter P. (**E**) The thermal adaptation of WT, *Atadf1*, *AtADF1*−OE, *Atmyb30*, *Atmyb30 Atadf1−1,* and *Atmyb30 AtADF1*−OE seedlings was visualized. Three−day−old seedlings of WT, *Atadf1*, *AtADF1*−OE, *Atmyb30*, *Atmyb30 Atadf1,* and *Atmyb30 AtADF1*−OE were transferred into a 28 °C chamber for four days. Scale bar = 1 cm. (**F**,**G**) Leaf area and fresh weight of WT, *Atadf1*, *AtADF1*−OE, *Atmyb30*, *Atmyb30 Atadf1* and *Atmyb30 AtADF1*−OE seedlings at normal (22 °C) and high temperature (28 °C). The number in the columns is the percentage increase between normal and high temperature conditions. At least 60 leaves from 30 seedlings were examined for leaf area, and at least 300 seedlings were examined for fresh weight, per genotype and treatment. Values are mean ± SD from three independent replicates. All of the statistical analyses use a one-way ANOVA followed by a Tukey’s post-hoc test. Significant differences were indicated by different lowercase letters.

A previous study reported that AtMYB30 is a key transcription factor in heat stress [31]. The RT-qPCR data indicated that the expression of *AtMYB30* was down-regulated by high temperature treatments (Figure S1), confirming that *AtMYB30* responded to high temperature. The T-DNA insertion mutant *Atmyb30* and *AtMYB30* overexpression (*AtMYB30* OE) transgenic line (*35S::GFP-AtMYB30*) were identified (Figure S2). Phenotypic analysis showed that the increase rate of leaf area and fresh weight of *Atmyb30* seedlings was higher than that of WT under high temperature conditions, but that of *AtMYB30* OE seedlings was opposite (Figure S3). To explore whether AtMYB30 regulates the transcription level of *AtADF1* under high temperature, the expression of *AtADF1* in WT, *Atmyb30,* and *AtMYB30* OE seedlings treated with 28 °C for 4 d was determined. The results showed that the expression of *AtADF1* in *AtMYB30* OE seedlings was significantly higher than that in WT under high temperature treatments (Figure 3A).

Further promoter analysis revealed that the promoter of *AtADF1* contained one putative AtMYB30 binding site (AACAAAC) [31,39] (Figure 3B). Chromatin immunoprecipitation (ChIP) assays were performed to confirm whether AtMYB30 was able to bind the AtMYB30 binding site of the *AtADF1* promoter. The fragment *AtADF1-N* (−167 to −70 region) [40] in the *AtADF1* promoter (translational start is +1), which did not contain an AtMYB30 binding site, and *AtACT7* were used as the negative control (Figure 3B). The fragment containing the AtMYB30 binding site in the *AtPIF4* promoter [30] was used as a positive control (Figure 3B). The ChIP-qPCR data indicated that the fragment *AtADF1-P* (−394 to −492 region) containing the AtMYB30 binding site in the *AtADF1* promoter was highly enriched in the anti-AtMYB30 but not in the mock controls (Figure 3C). Then, electrophoretic mobility shift assay (EMSA) results showed that the GST-MYB30 fusion protein could bind to the fragment *AtADF1-P* rather than the fragment *AtADF1-N* of the *AtADF1* promoter (Figure 3D). The biotin binding signals decreased with the increasing competition from cold probes (Figure 3D). Together, these results demonstrated that AtMYB30 directly binds to the *AtADF1* promoter in vivo and in vitro.

To further test the function of AtMYB30 regulating *AtADF1* under high temperature, we created *Atmyb30 Atadf1* and *Atmyb30 AtADF1*-OE hybrid materials (Figure S4). Under high temperature conditions, the increase rate of leaf area and fresh weight of *Atmyb30 Atadf1* double mutant seedlings was greater than that of WT, while *Atmyb30 AtADF1*-OE seedlings had opposite phenotypes (Figure 3E–G), which was consistent with the phenotypes of *Atadf1-1* and *AtADF1*-OE#33 seedlings. These results indicated that *AtADF1* is the direct target gene of AtMYB30, participating in plant growth under high temperature conditions.

### 2.4. Sequence Analysis of Chinese Cabbage ADF1 (BrADF1)

**Figure 4:**
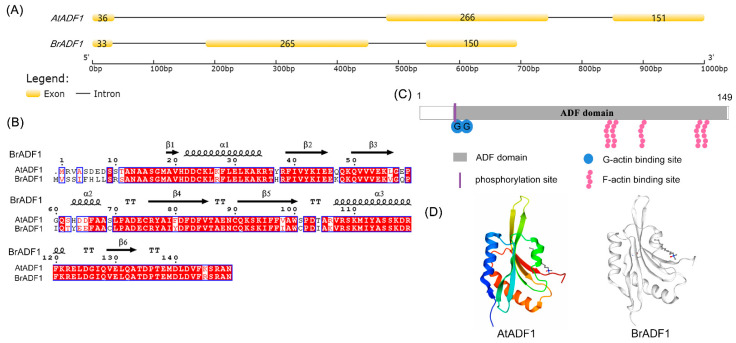
Sequence and structure analysis of Chinese cabbage ADF1 (BrADF1). (**A**) Gene structure alignment of *AtADF1* and *BrADF1*. Gene structures contain three exons and two introns in *AtADF1* and *BrADF1*. (**B**) Protein sequence alignment of AtADF1 and BrADF1. The top is the predicted secondary structure of protein BrADF1. (**C**) Predicted domain architecture of BrADF1. The BrADF1 coding sequence encodes 150 amino acids containing one ADF domain (amino acids 16–149), one phosphorylation site (amino acid 10), two G-actin binding sites (amino acids 16 and 17), and five F-actin binding sites (amino acids 92, 94, 108, 135, and 138). (**D**) Predicted tertiary structure of BrADF1, which is similar to the tertiary structure model 1F7S of AtADF1.

High temperatures affect the yield of Chinese cabbage. At present, there are few reports on the adaptation of Chinese cabbage to high temperatures, especially the role of actin filaments in Chinese cabbage adaptation to high temperatures. The gene structure of Chinese cabbage *BraA02g010580.3C* (*BrADF1*) was similar to *AtADF1*, including three exons and two introns (Figure 4A). BrADF1 contained a 450 bp open reading frame encoding a protein of 150 amino acids. The similarity of the protein sequence and coding region between BrADF1 and AtADF1 was 81.33% and 79.25%, respectively (Figure 4B). The molecular weight of BrADF1 is 16.975 kDa, and the theoretical isoelectric point (pI) is 5.26. Further analysis showed that BrADF1 may contain one phosphorylation site (amino acids 10), two G-actin binding sites (amino acids 16 and 17), and five F-actin binding sites (amino acids 92, 94, 108, 135, and 138) (Figure 4C). In addition, homology modeling of the BrADF1 protein showed that the predicted tertiary structure was highly similar to AtADF1, and the tertiary structure model was 1F7S (Figure 4D). These data suggested that the potential functions between BrADF1 and AtADF1 were similar.

### 2.5. BrADF1 Inhibits Plant Growth and Actin Filaments Stability under High Temperature

**Figure 5:**
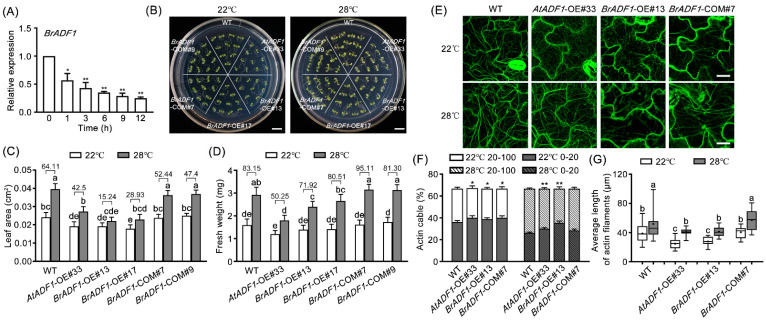
BrADF1 is a negatively regulator in plant growth and the stability of actin filaments under high temperature. (**A**) The relative expression of *BrADF1* in 7-day-old Chinese cabbage “DH” line “FT” seedlings treated at 28 °C for 1, 2, 4, and 6 d was determined by RT-qPCR. Chinese cabbage *ACTIN* was used as an internal control. Values are means ± SD from three independent replicate experiments (Student’s *t*-test, * p < 0.05, ** p < 0.01). (**B**) The thermal adaptation of WT, *AtADF1*-OE#33, *BrADF1*-OE#13, *BrADF1*-OE#17, *BrADF1*-COM#7, and *BrADF1*-COM#9 was visualized. Scale bar = 1 cm. (**C**,**D**) Leaf area and fresh weight of WT, *AtADF1*-OE#33, *BrADF1*-OE#13, *BrADF1*-OE#17, *BrADF1*-COM#7, and *BrADF1*-COM#9 seedlings at normal (22 °C) and high (28 °C) temperatures. At least 60 leaves from 30 seedlings were examined for leaf area, and at least 300 seedlings were examined for fresh weight, per genotype and treatment. Values are mean ± SD from three independent replicates. All of the statistical analyses use a one-way ANOVA followed by a Tukey’s post-hoc test. Significant differences were indicated by different lowercase letters. The number in the column is the percentage increase between normal and high temperature conditions. (**E**) Representative images of the actin filaments in leaf pavement cells of WT, *AtADF1*-OE#33, *BrADF1*-OE#13, and *BrADF1*-COM#7 seedlings grown at 22 °C and 28 °C. Scale bar = 25 μm. (**F**) The fluorescence intensity of actin cables. In the same condition, statistical analysis revealed a significant difference between WT (0–20 and 20–100) and genotypes (0–20 and 20–100). Values are means ± SD from three independent replicate experiments (n > 300 images from at least 30 seedlings per genotype and treatment). Student’s *t*-test, * p < 0.05, ** p < 0.01). (**G**) Average length of actin filaments in WT, *AtADF1*-OE#33, *BrADF1*-OE#13, and *BrADF1*-COM#7 seedlings grown at 22 °C and 28 °C. Values are means ± SD from three independent replicate experiments. All of the statistical analyses use a one-way ANOVA followed by a Tukey’s post-hoc test. Significant differences were indicated by different lowercase letters.

To distinguish whether BrADF1 responds to high temperature, we analyzed the expression pattern of *BrADF1* under high temperature treatments. The RT-qPCR results showed that the transcript level of *BrADF1* was markedly reduced with high temperature treatments at 4 and 6 d (Figure 5A), indicating a *BrADF1* response to high temperature that was similar to *AtADF1*. Next, we constructed *BrADF1*/WT overexpression (*BrADF1*-OE) lines and *BrADF1/Atadf1-1* complementation (*BrADF1*-COM) lines in *Arabidopsis* (Figure S5). As shown in Figure 5B, under high temperature conditions, the reduction rate of leaf area and fresh weight of *BrADF1*-OE#13 and *BrADF1*-OE#17 seedlings was lower than that of WT, while the leaf area and fresh weight of *BrADF1*-COM#7 and *BrADF1*-COM#9 seedlings were similar to WT (Figure 5B–D), which was similar to the phenotypes of *AtADF1-*OE#33 and *AtADF1-*COM seedlings. These results demonstrated that BrADF1 negatively regulated Arabidopsis growth under high temperature conditions, suggesting that BrADF1 and AtADF1 have the same role in plant thermal adaptation.

*BrADF1-GFP/Atadf1-1* transgenic plants were constructed to investigate the activity of BrADF1 in binding to actin filaments in vivo. The results showed long filaments existed in pavement cells of leaves, and the filament structures were disrupted into short filaments after 50 nM Latrunculin B (LatB, a microfilament-depolymerizing drug) treatment (Figure S6). Contrary to LatB treatment, the filament structures were more stable than the control after 1 μM Phalloidin (a microfilament stabilization drug) treatment and had longer and thicker filaments (Figure S6). Moreover, the filament structures were not changed by 50 nM Oryzalin (a microtubule-depolymerizing drug) treatment, confirming that BrADF1 specifically binds to actin filaments in *Arabidopsis* (Figure S6).

To evaluate the function of BrADF1 in regulating the dynamics of actin filaments, the actin filament organization of *BrADF1*-OE#13 and *BrADF1*-COM#7 seedlings fused with *f*ABD2-GFP was observed (Figure 5E). The intensity of fluorescence of actin cables, representing the degree of stability of actin filaments, was measured according to the methods reported by Zou et al. [41]. Low (0–20 pixels) and high (20–100 pixels) fluorescence intensities representing weakly and strongly fluorescent actin cables were defined by previous reports [20,40,41,42]. Compared with WT, the actin cables at low fluorescence intensities were significantly increased in *AtADF1*-OE#33 and *BrADF1*-OE#13 seedlings, and there was no significant difference in *BrADF1*-COM#7 seedlings under high temperature conditions (Figure 5F). Furthermore, the average length of actin filaments of *AtADF1*-OE#33 and *BrADF1*-OE#13 seedlings was significantly shorter than that of WT under the normal and high temperature conditions, and *BrADF1*-COM#7 seedlings were similar to WT (Figure 5G). These results demonstrated that BrADF1 inhibited the stability of actin filaments under high temperatures in *Arabidopsis*, suggesting that BrADF1 regulates plant growth by affecting the organization of actin filaments.

## 3. Discussion

### 3.1. AtADF1, Mediated by AtMYB30, Plays a Negative Role in Plant Thermal Adaptation by Affecting Actin Filament Organization

Plant exposure to a high temperature environment results in a series of changes in gene expression, therefore improving plant thermo-tolerance [26,37,43,44,45]. Previous reports showed that ADFs play a key role in multiple abiotic and biotic stresses during plant development [18]. Huang et al. found that the expression of *OsADF3* is induced by salt and drought stress, and the overexpression of *OsADF3* confers Arabidopsis drought tolerance [17]. In addition, *Arabidopsis* AtADF5 promotes stomatal closure in response to ABA and drought stress [19]. It is reported that AtADF1 is widely expressed in plant tissues and participates in salt stress by affecting actin filament rearrangement [21,25,40]. Here, we explored the role of AtADF1 during plant adaptation to high temperatures. In this study, we found that high temperatures repressed the transcription of *AtADF1* (Figure 1). Compared with the seedlings grown at 22 °C, the seedlings transferred to 28 °C showed significantly more rapid growth (Figure 2A). *Atadf1-1* mutant seedlings showed a higher growth rate than WT seedlings at 28 °C; however, the *AtADF1*-overexpression line had the opposite phenotype (Figure 2B,C), suggesting that AtADF1 is a negative regulator in thermal adaptation in *Arabidopsis*.

High temperatures affect plant growth, development, and agricultural productivity [8]. It is reported that actin filaments are involved in thermal response [2,7,8,11]. Besides a few thick and short actin bundles remaining in root epidermal cells, strong diffuse background fluorescence was visible after 41 °C treatment for 10 min. However, thick bundles were formed after plants were exposed to 35 °C for 6 h [10]. Consistent with this result, we observed the bundled actin filaments in leaf pavement cells of seedlings marked by *fABD2*-GFP at high temperatures. The seedlings grew on 1/2 MS for 3 d, then were transferred to 28 °C for another 4 d. High temperature induced actin filament assembly and bundle formation, suggesting that the stabilization of actin filaments is required for plant adaptation to high temperatures. In addition, we found that the actin filaments in *Atadf1-1* mutant seedlings had higher skewness and lower density than those in WT seedlings under the same conditions (Figure 2E,F), demonstrating that the deletion of AtADF1 enhances the stability of actin filaments and leads to an improvement in Arabidopsis thermal adaptability. The function of AtADF1 is to depolymerize/sever actin filaments [15,21,46]. AtADF1 plays a role in the depolymerization of actin filaments under high temperature, which is consistent with the result under salt stress [40], suggesting that different environmental signals may not affect the property of AtADF1 in depolymerizing actin filaments. Furthermore, the expression of *AtADF1* was down-regulated, and the leaf area of *Atadf1-1* seedlings was larger than WT under high temperature conditions, indicating that AtADF1 acts as a negative regulator (Figure 1 and Figure 2). However, AtADF1 plays a role as a positive regulator under salt stress [40]. The difference may be related to the fact that plants produce different dynamics of actin filaments under different stresses or at different times under the same stress, resulting in different physiological functions [40,47]. Therefore, it is necessary to deeply understand the physiological functions of AtADF1 under various environmental conditions.

**Figure 6:**
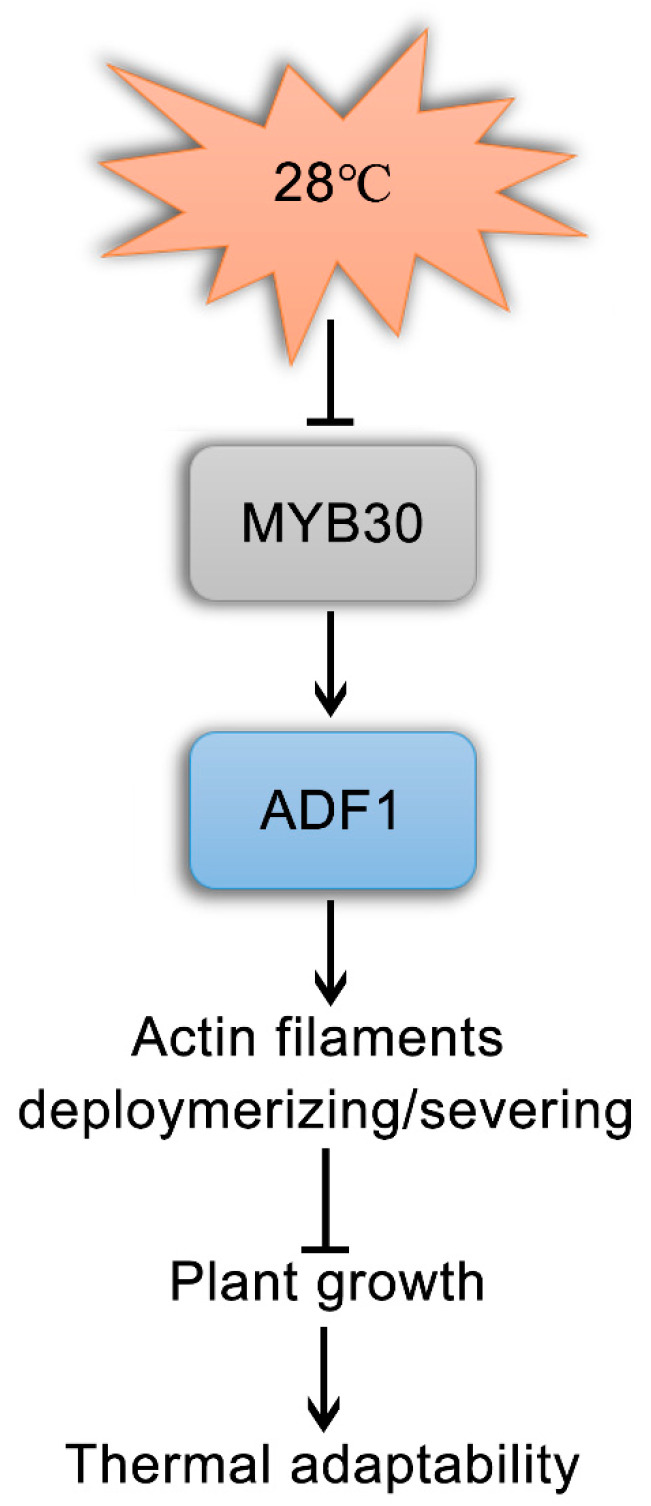
Working model of AtMYB30 and AtADF1 in *Arabidopsis* plants in response to high temperatures. Arrows represent positive regulation, and barred ends indicate inhibitory action. Details of this model are discussed in the text.

Previous studies have reported that AtADF1 is involved in post-transcriptional regulation [40,48]. Additionally, we recently found that AtADF1 is a target gene of AtMYB73 in plant response to salt stress [40]. AtMYB73 only responds to salt stress among various abiotic stresses [40,49], so AtADF1 may be regulated by other transcription factors under high temperature conditions. It is reported that *AtMYB30,* as an important negative regulatory transcription factor, is involved in high temperatures [30,31]. In this study, we proved that AtMYB30 binds to the promoter of *AtADF1* at the MYB-binding site AACAAAC (Figure 3B–D). In addition, the expression of *AtADF1* in *AtMYB30* OE seedlings was promoted, and the phenotypes of *Atmyb30 Atadf1-1* and *Atmyb30 AtADF1*-OE#33 were consistent with those of *Atadf1-1* and *AtADF1*-OE#33 seedlings under high temperature conditions (Figure 3A,E), demonstrating that *AtADF1* is a downstream target gene of AtMYB30 and promoted by AtMYB30 to negatively regulate plant thermal adaptation (Figure 6).

### 3.2. BrADF1 Plays a Similar and Important Role to AtADF1 in Plant Thermal Adaptation

High temperatures significantly affect the growth and development of Chinese cabbage [33]. However, the dynamics of microfilaments and their molecular mechanisms in Chinese cabbage thermal adaptation are still unclear. Therefore, in the present study, we investigated the role of Chinese cabbage BrADF1 under high temperatures. We identified that the sequence of *BrADF1* was highly similar to *AtADF1* (Figure 4). Further studies showed that *BrADF1*-overexpression seedlings had smaller leaf areas and lighter fresh weights than WT, and the *BrADF1* complementation line rescued the AtADF1 deletion phenotypes (Figure 5C,D), which were consistent with *AtADF1*-overexpression lines. Additionally, BrADF1 decorated actin filaments in the pavement cells of *Arabidopsis* leaves (Figure S6). Meanwhile, the ratio of actin cables with high fluorescence intensity and the average length of actin filaments significantly reduced in *AtADF1*-OE#33 and *BrADF1*-OE#13 seedlings (Figure 5F,G), demonstrating that BrADF1 depolymerizes actin filaments and inhibits microfilament stabilization under high temperature, which is similar to AtADF1. These results indicated that Chinese cabbage BrADF1 and *Arabidopsis* AtADF1 play a similar key role in responding to high temperatures.

There are three *AtMYB30* homologous genes in Chinese cabbage, including BraA02g038430.3C, BraA06g036730.3C, and BraA09g003630.3C. Sequence analysis showed that all BrMYB30s contained the same MYB binding domain as AtMYB30 (Figure S7). In addition, the promoter of *BrADF1* contains the other two MYB30 binding sites, TATCC and TTTGGTT, which were reported by Liao et al. (2017), suggesting that BrMYB30s may be involved in *BrADF1*-mediated adaptation to high temperatures. However, which form of BrMYB30 binds to the *BrADF1* promoter needs further study. In summary, we identified that *AtADF1* is regulated by the R2R3-MYB transcription factor AtMYB30 to maintain the stability of the actin filaments and improve plant thermal adaptation, while BrADF1 possesses the same function. These results provide breakthrough evidence for understanding the regulation of microfilaments in plant thermal adaptation.

## 4. Materials and Methods

### 4.1. Plant Materials, Growth Conditions, and Heat Treatments

In this study, all ecotypes of *Arabidopsis thaliana* plants have a Columbia background. The T-DNA insertion mutant *Atadf1-1* was obtained from ABRC. *AtADF1* coding sequence and *pAtADF1::AtADF1* sequence were cloned into the *pCAMBIA1300-221* vector to generate *35S::AtADF1* and *pAtADF1::AtADF1*, which were then transformed into WT and *Atadf1-1,* respectively, by Agrobacterium-mediated transfection [40]. The plants fused with *fABD2-GFP* and *pADF1::GUS* plants were built in the previous reports [40]. The T-DNA insertion *Atmyb30* mutant (SALK_122884) was obtained from ABRC. The primers (*Atmyb30* LP/RP) were used for identifying homozygotes listed in Table S1. Chinese cabbage “DH” line “FT” seeds were provided by professor Hui Feng.

All Arabidopsis seeds were vernalized at 4 °C for 2 days. Then these seeds were sown on one-half strength Murashige and Skoog (1/2 MS) medium, whose pH was adjusted to 5.8 with 1 M KOH and which contains 1.5% (*w*/*v*) sucrose and 0.8% (*w*/*v*) agar. The plated seeds were grown in a 22 °C chamber in 16 h light/8 h dark conditions. For thermal adaptation analysis, 3-day-old seedlings growing at 22 °C were transferred to a 28 °C chamber for 4 days.

### 4.2. Gene Expression and GUS Activity Analysis

To assay the expression of *AtADF1* under heat treatment in *Arabidopsis*, 3-day-old wild-type seedlings treated at 28 °C for 1, 2, 3, and 4 d were collected. 7-day-old Chinese cabbage plants were placed in a 28 °C chamber for 1, 2, 4, and 6 d, then the leaves were collected to extract total RNA. To examine the expression of *AtADF1* in WT, *Atmyb30,* and *AtMYB30* OE seedlings and the expression of heat response genes in WT, *Atadf1-1*, *AtADF1*-OE#33, and *BrADF1*-OE#13 seedlings, 3-day-old seedlings were treated at 28 °C for 4 d and collected for further experiment. All total RNA was extracted using the Easy Pure Plant RNA kit from TransGen Biotech Company. The relative expression levels of *AtADF1* were detected by Roche LightCycler 480. All RT–qPCR results were performed with three independent biological replicates. *18S* and *ACTIN* were used as internal controls in *Arabidopsis* and Chinese cabbage. Primers are listed in Table S1.

To analyze the GUS activity of *AtADF1* under heat treatments in *Arabidopsis*, 3-day-old *pADF1:GUS* seedlings were treated at 28 °C for 1, 2, 3, and 4 d. The whole plants were observed to analyze the changes in GUS activity. Each experiment was repeated at least three times with similar results.

### 4.3. Chromatin Immunoprecipitation-Quantitative PCR (ChIP-qPCR) Assay

The full-length coding sequence (CDS) of *AtMYB30* was amplified and cloned into the pCAMBIA1205-GFP vector with an N-terminal GFP. The primers used for cloning (*AtMYB30 CDS*-F/R) are listed in Supplementary Table S1. *35S::GFP-AtMYB30* was transformed into WT by *Agrobacterium*-mediated transfection using the floral dip method [50]. The 10-day-old *35S::GFP-AtMYB30* (*AtMYB30* OE) T_4_ transgenic plants were used for the ChIP assay with previously described methods [51]. The anti-GFP antibody was used. Both immunoprecipitated DNA and input DNA were examined by RT–qPCR. The primers used for ChIP (*AtADF1*-P-F/R or *AtADF1*-N-F/R) are listed in Supplementary Table S1. The primers of the fragment in the *AtPIF4* promoter containing the AtMYB30 binding site [30] used for positive control are listed in Table S1.

### 4.4. Electrophoretic Mobility Shift Assays (EMSA)

The 1-477 bp coding sequence of *AtMYB30* [31] was amplified and cloned into the pGEX4T-1 vector with glutathione S-transferase (GST). GST-AtMYB30 fusion proteins were induced in *Escherichia coli* BL21. The induction condition of *Escherichia coli* cells was described in a previous study [31]. The primers used for cloning (*AtMYB30* CDS 1-477 bp-F/R) are listed in Supplementary Table S1. The purification method of GST-AtMYB30 fusion protein and the EMSA experiment method were described by Wang et al. (2021). The fragment *AtADF1-P* of the *AtADF1* promoter with biotin-labeling and the fragment *ADF1-N* with unlabeled DNA were obtained by PCR. The primers used for EMSA are the same as those for ChIP.

### 4.5. Sequence Analysis of BrADF1

The gene sequence, CDS sequence, and amino acid sequence of BrADF1 were identified from the Brassica database using the Blast tool (http://brassicadb.cn/#/ (accessed on 16 October 2019)). The alignment of gene structure was displayed by the Gene Structure Display Server (http://gsds.gao-lab.org/ (accessed on 16 October 2019)). The crystal structure of AtADF1 was downloaded from the RCSB PDB (http://www.rcsb.org/ (accessed on 16 October 2019)). Multiple sequence and protein structure alignments were performed using CLUSTALW (https://www.genome.jp/tools-bin/clustalw (accessed on 16 October 2019)) and ESPript (http://espript.ibcp.fr/ESPript/cgibin/ESPript.cgi (accessed on 16 October 2019)). The molecular weight and theoretical pI of BrADF1 were calculated using ProtParam (http://web.expasy.org/protparam/ (accessed on 16 October 2019)). The protein domain predictions were assigned based on analysis using the SMART database (http://smart.embl-heidelberg.de/ (accessed on 16 October 2019)). The tertiary structure of BrADF1 was predicted by SWISS-MODEL (https://swissmodel.expasy.org/interactive (accessed on 16 October 2019)).

### 4.6. Plasmid Construction and Plant Transformation

450-bp *BrADF1* CDS fragments were cloned into the pCAMBIA1300-211 and pCAMBIA1205-GFP vectors to construct *35S::BrADF1* and *35S::BrADF1-GFP*.

The primers used for plasmid construction (*BrADF1* CDS-F/R) are listed in Table S1. All plasmids were introduced into the *Agrobacterium tumefaciens* strain GV3101. *35S::BrADF1* were transformed into WT and *Atadf1-1* as *BrADF1*-OE and *BrADF1*-COM and *35S::BrADF1-GFP* was transformed into *Atadf1-1* (*BrADF1-GFP/Atadf1-1*) by Agrobacterium-mediated transfection using the floral dip method. All transgenic plants were selected on ½ MS medium supplied with hygromycin for homozygotes. Genetically modified materials from the *BrADF1*-OE#13, *BrADF1*-OE#17, *BrADF1*-COM#7, and *BrADF1*-COM#9 lines were selected for further study. The primers used for RT-qPCR (*BrADF1* RT-qPCR -F/R) are listed in Table S1.

### 4.7. Visualization and Analysis of Actin Filament Architecture and the Subcellular Localization of BrADF1

Arabidopsis *Atadf1-1* and *AtADF1*-OE#33 plants that hybridized with *f*ABD2-GFP wild-type were described in previous reports. *BrADF1*-OE#13 and *BrADF1*-COM#7 seedlings were hybridized with *f*ABD2-GFP wild-types to visualize the actin filaments. *35S::BrADF1-GFP/Atadf1-1* was visualized using the location of BrADF1. A laser scanning confocal microscope with a ×40 objective was used to observe actin filament morphology with a 488-nm laser.

As described in previous reports, skewness, density, and the actin cables were measured in Image J (GPL v3.0) [20,41]. Skewness indicates the degree of actin bundles, while density is a measure of the abundance of actin filaments. The experiment was repeated three times. At least 300 cells from 60 images were collected from 30 individual seedlings for each genotype and treatment.

### 4.8. Western Blot Assays

The *pADF1::ADF1* fragment was constructed in the *pSupper1300-GFP* vector to obtain *pADF1::ADF1-GFP/adf1-1* transgenic plants (*AtADF1*-GFP) using previous methods [40]. 10-day-old *AtADF1*-GFP seedlings were treated at 28 °C for 1, 2, 3, and 4 d. GFP, as a labeled protein, was analyzed by SDS-PAGE as described in previous reported protocols [52,53]. Rubisco bands were used as loading controls.

### 4.9. Statistical Analysis

Mean values, SD, and SE were calculated with Microsoft Excel software. Statistical analysis of relative expression was conducted using an independent sample *t*-test (* *p* < 0.05, ** *p* < 0.01, *** *p* < 0.001). Other data was analyzed using a one-way ANOVA implemented in the SPSS software, followed by a Tukey’s post-hoc test and least significant difference (LSD) test at a significance level of *p* < 0.05. Significant differences were indicated by different lowercase letters.

## Data Availability

All data are presented in article and Supplementary Materials.

## Supplementary Materials

The following supporting information can be downloaded at: https://www.mdpi.com/article/10.3390/ijms24065675/s1.
