# Supplementary figures and images for: Actin Depolymerization Factor ADF1 Regulated by MYB30 Plays an Important Role in Plant Thermal Adaptation
Source: Int J Mol Sci. 2023 Mar 16;24(6):5675. doi: 10.3390/ijms24065675 (PMC10051699; doi:10.3390/ijms24065675)

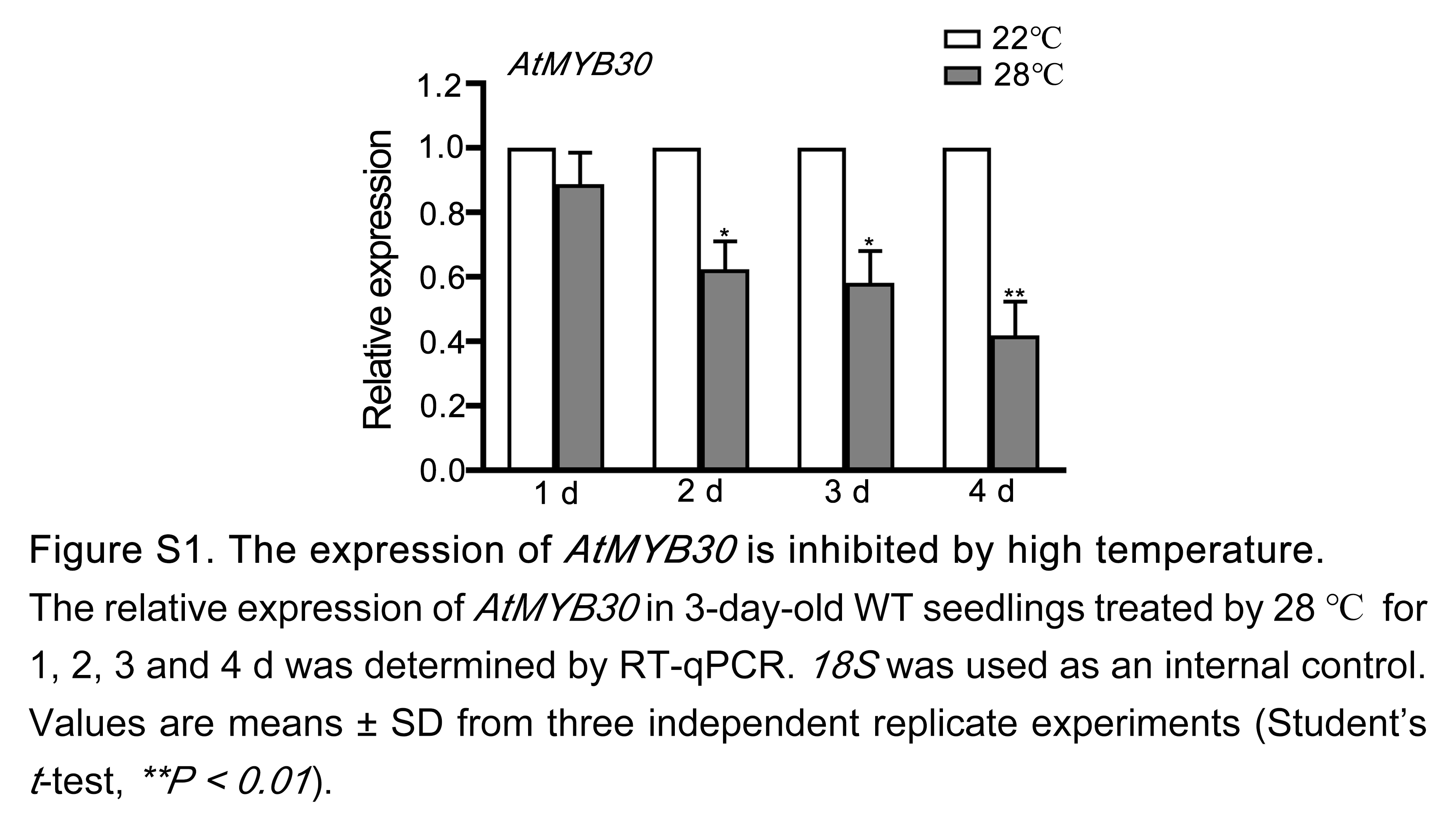

Supplement: Supplementary file 1 [file ijms-24-05675-s001.zip › ijms-2180622-supplementary/Figure S1.tif]

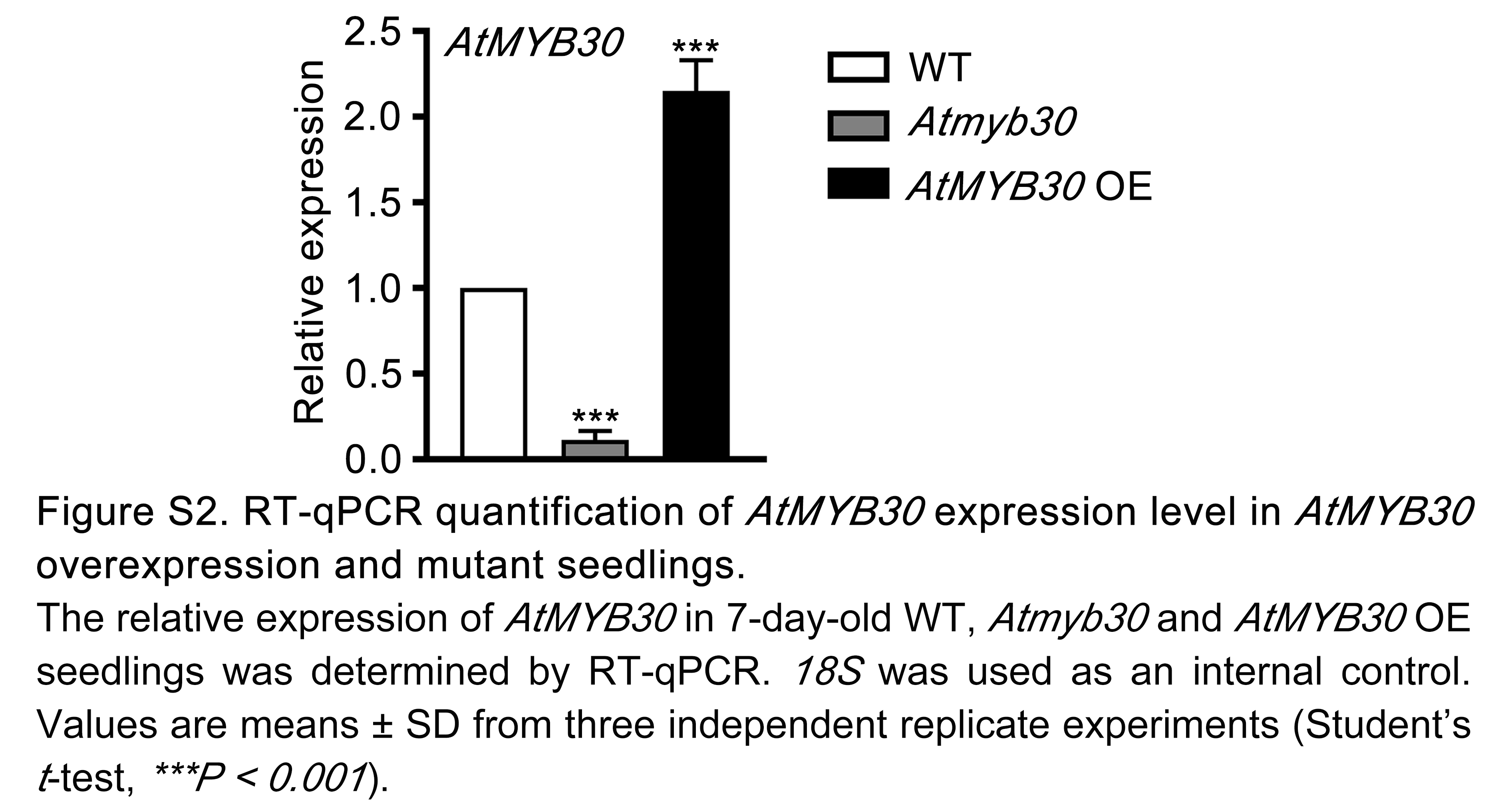

Supplement: Supplementary file 1 [file ijms-24-05675-s001.zip › ijms-2180622-supplementary/Figure S2.tif]

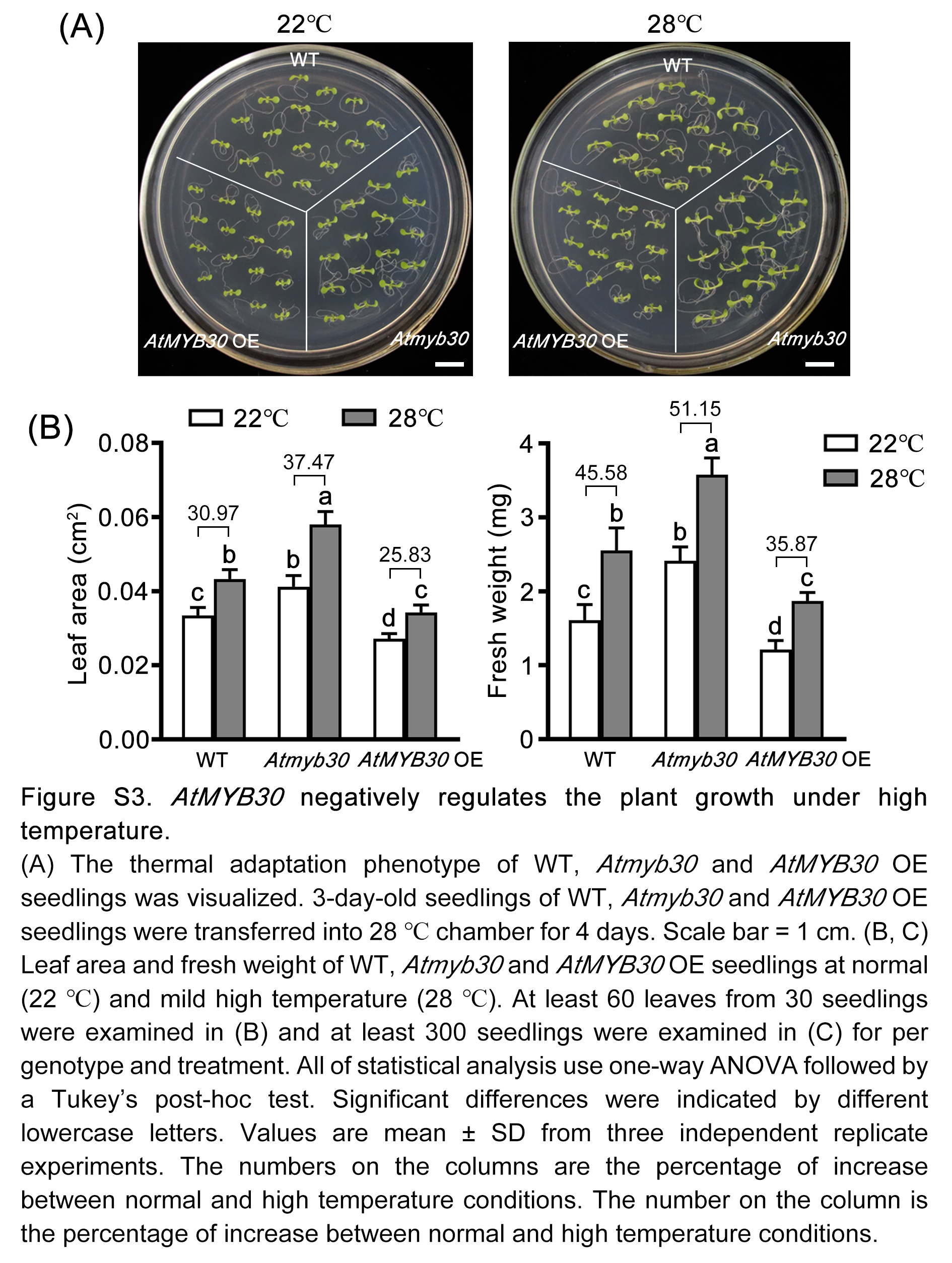

Supplement: Supplementary file 1 [file ijms-24-05675-s001.zip › ijms-2180622-supplementary/Figure S3.tif]

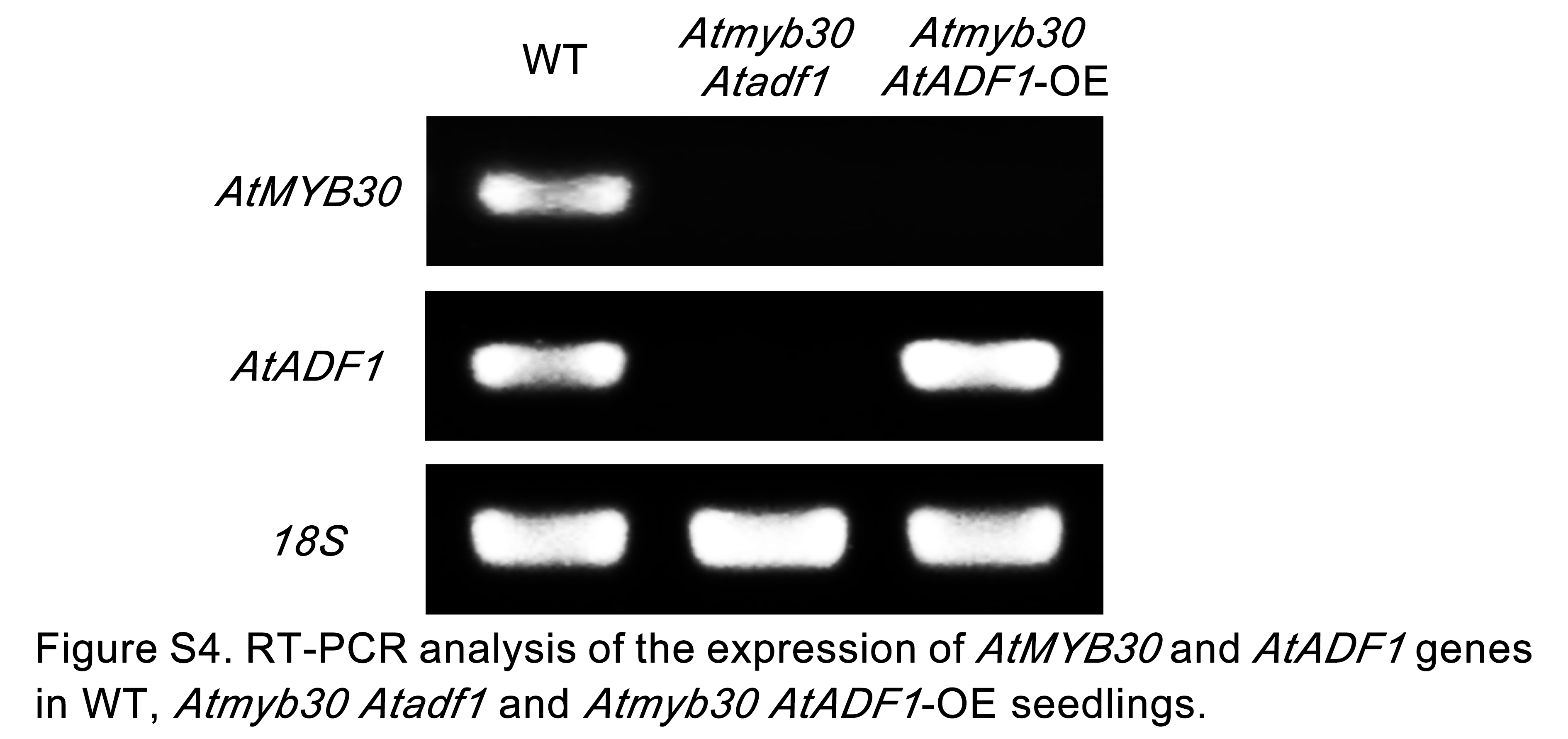

Supplement: Supplementary file 1 [file ijms-24-05675-s001.zip › ijms-2180622-supplementary/Figure S4.tif]

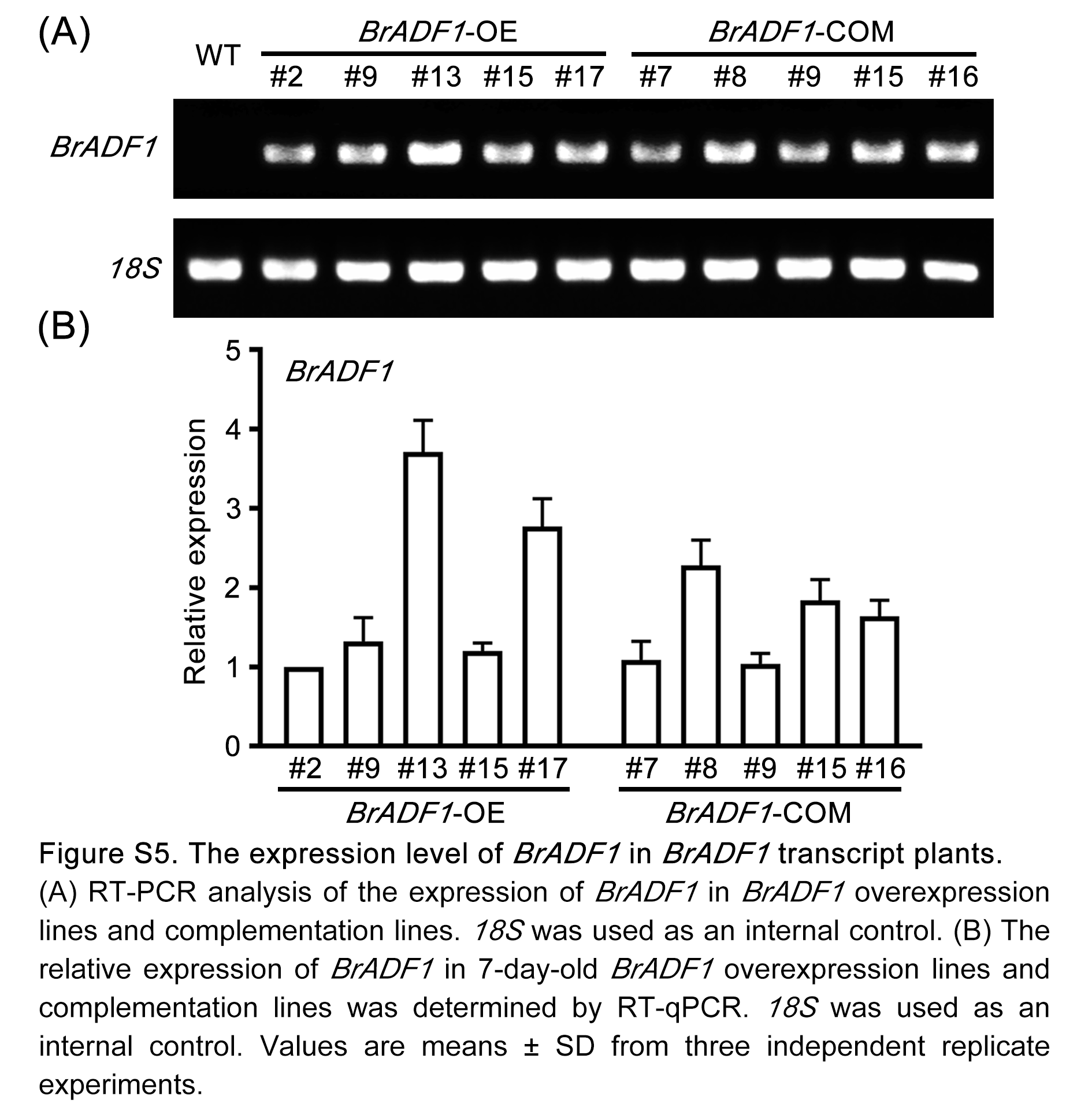

Supplement: Supplementary file 1 [file ijms-24-05675-s001.zip › ijms-2180622-supplementary/Figure S5.tif]

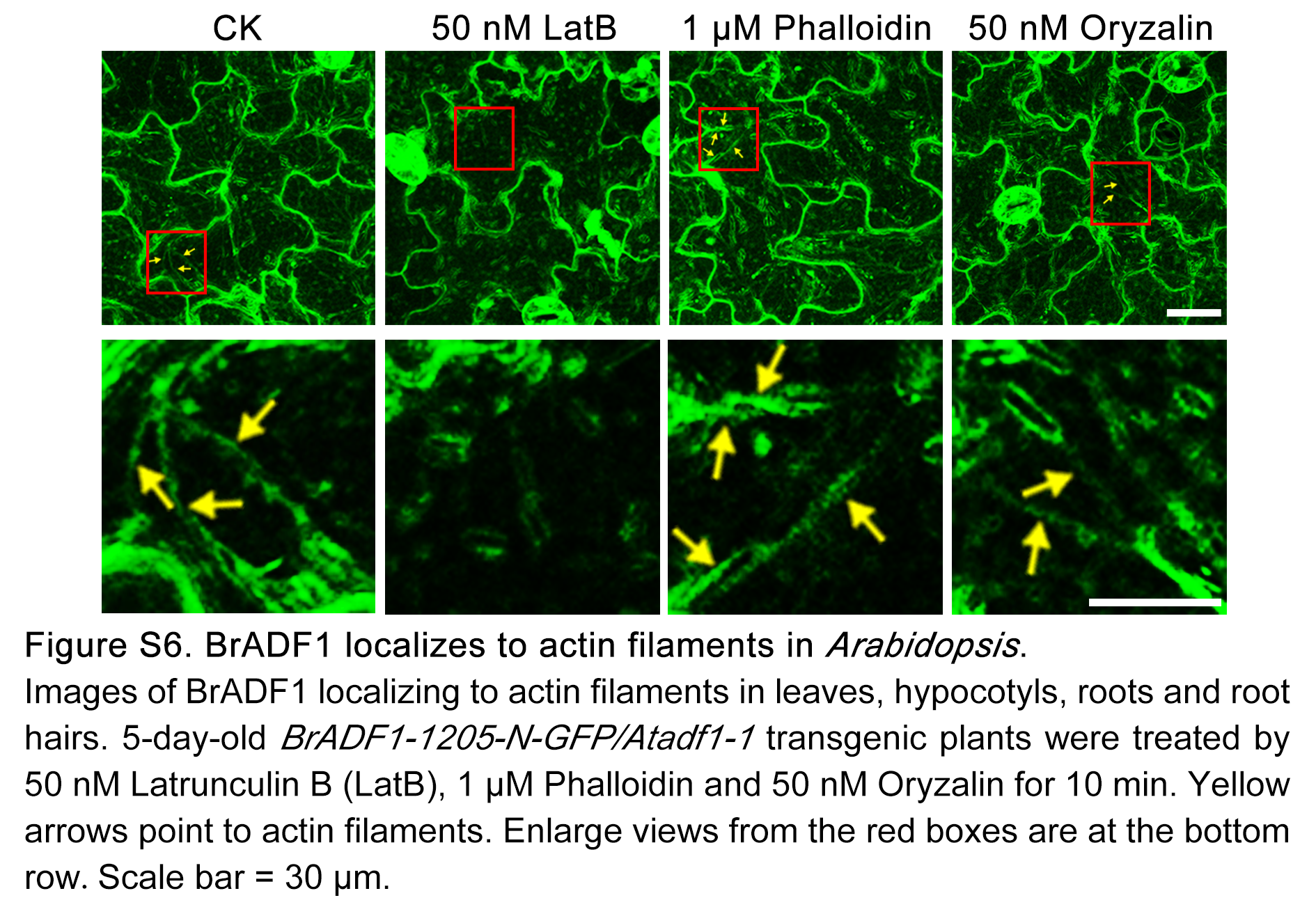

Supplement: Supplementary file 1 [file ijms-24-05675-s001.zip › ijms-2180622-supplementary/Figure S6.tif]

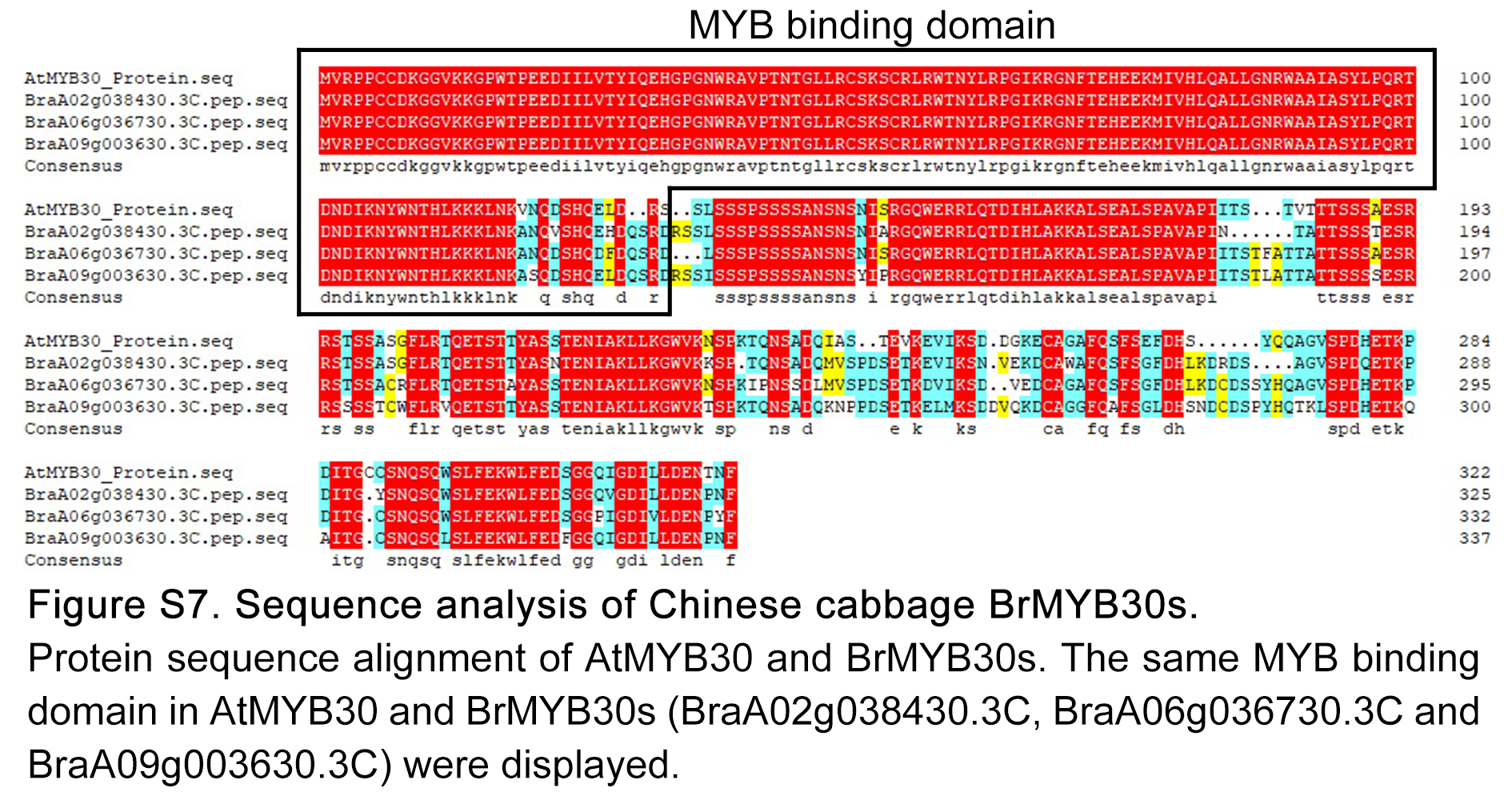

Supplement: Supplementary file 1 [file ijms-24-05675-s001.zip › ijms-2180622-supplementary/Figure S7.tif]
